# A New Method of Whitening Bones

**Published:** 1852-01

**Authors:** 


*A New Method of Whitening Bones.* By Ellerslie Wallace, M. D., Demonstrator of Anatomy in Jefferson Medical College, Philadelphia.

To the Editors of the Medical Examiner:

*Gentlemen:*—During the past year, I have used sulphuric ether for the purpose of extracting the greasy matters from bones of which I have desired to make preparations, and have uniformly found it entirely satisfactory. I have used it for entire skeletons where they have been of value.

Twenty-five or thirty pounds of ether (which can be obtained for 18 cts. per lb.) is enough for a skeleton, if the bones be closely packed in a proper case. After pouring the ether on them until they are entirely covered, they may be left for some hours, or a day; then removing them, they should be allowed to dry thoroughly. This process should be repeated as often as may be necessary.

Six immersions have been enough for a very greasy skeleton. It is prudent to wash the ether before using it, to remove free acid, and we may have the ether re-distilled after it is saturated with oil. To morbid specimens, as of caries, &c., it is admirably adapted, as it removes the grease entirely, without injuring the delicate structure at all; which is not the case, as we all know, with any of the ordinary alkaline solutions.

Very truly, yours, ELLERSLIE WALLACE.

92 *S.* 11*th St., Nov.* 1*st*, 1851.

